# Child Wantedness and Low Weight at Birth: Differential Parental Investment among Roma

**DOI:** 10.3390/bs10060102

**Published:** 2020-06-18

**Authors:** Jelena Čvorović

**Affiliations:** Institute of Ethnography, SASA, 11 000 Belgrade, Serbia; jelena.cvorovic@ei.sanu.ac.rs

**Keywords:** child wantedness, birthweight, bias parental investment, Roma

## Abstract

Studies investigating child wantedness, birthweight and parental care are limited. This study assessed relationships of child wantedness, low birthweight and differential parental investment in a poor population of Serbian Roma. Data from the Multiple Indicator Cluster Survey round 5 for Roma settlements were used to account for the association between child wantedness and birthweight, and three measures of parental investment: breastfeeding practices, immunization of children and quality of mother–child interaction. The sample included 584 children aged 0–24 months. The child variables were gender, birth order, birthweight (low birthweight at <2500 g and normal birthweight at >2500 g) and whether the child was wanted, while maternal independent variables included age, literacy and household wealth. The results show that unwanted children were at greater risk of having low birthweight. After controlling for birthweight, child wantedness emerged as a predictor of breastfeeding practices and immunization status: Roma mothers biased their investment toward children who were wanted. The quality of mother–child interaction varied with the mother’s household wealth. Given the high rates of infant and child mortality among Roma, investments in children’s health should be prioritized within the family, where maternal bias in parental investment may contribute to their children’s health disparities.

## 1. Introduction

Parenting and caregiving practices may be the main determinant of child development, including impact on health outcomes [1,2]. Poverty affects parental conditions and may influence overall parental investment and how biased that investment is [3,4]. The relationship between poverty, parenting effort, ethnicity, and child outcomes in ethnic minority populations is poorly understood; yet, it may have important implications for programs aimed at improving the status of children living in poverty [5]. To address this aspect, this study examined bias in parental investment among poor population of Serbian Roma.

The Roma people are the largest ethnic group in Europe with low levels of integration, high rates of extreme poverty, marginalization, discrimination and poorer health compared to non-Roma [6]. Across Europe, the infant and child mortality rate of Roma children is much higher in comparison with the child mortality rate of the overall population of any given European country [7]. The disparity may be related to Roma infants having the highest prevalence of low birthweight, generally more than double that of non-Roma. The limited studies that have been published suggested that Roma ethnicity is independently associated with lower birthweight among at term neonates, and this difference remains even after controlling for known risk factors [8,9]. Birthweight reflects maternal size, reproductive strategy, and environmental limitations [10,11], but one important factor to consider when investigating birth differentials is unwanted childbearing. Births resulting from unwanted childbearing are the result of unintended pregnancies, further classified as unwanted pregnancies (occurring when no more children were wanted) or mistimed pregnancies (occurring at a different time than desired) [12]. Unwanted childbearing is considered an important public health problem, associated with adverse maternal behavior during pregnancy and poor perinatal outcomes such as low birthweight and infant mortality; the effects of unwanted childbearing resulting in a live birth (child wantedness) on a child’s care have not been extensively studied, while the existing research produced contradictory results [12,13,14,15,16].

The extent of parental investment is crucial for offspring survival and development, but parents tend to differentiate their investment toward offspring depending on the environment and the characteristics of both child and mother [17,18]. Under poor conditions and at high environmental risk, it is expected that there may be limited differential investment between children, as parents may have little control of their children’s survival and reproductive chances [19]. A previous study suggested that Roma mothers appear to compensate for handicaps associated with low birthweights and high mortality by having a larger number of closely spaced children with reduced amounts of parental investment [20]. Maternal investment starts in utero and lower birthweight is one of the key indicators of lower maternal investment during pregnancy [21]. As low-weight at birth is also a common proxy for health, it is expected to strongly influence a child’s reproductive value and parents may choose to shift their postnatal investments accordingly [22]. Another possible explanation for differential investment may be differences in child wantedness. If the child was unwanted, it may inflict threats on the health and wellbeing of the child, as the mother may perceive the child as an additional cost, a burden for which she is not prepared, and thus, may invest in the child less either because of resources or some other constraints [23]. Furthermore, the level of parental care may be of particular importance for children coming from the most disadvantaged backgrounds [24].

Over 140,000 Roma live in Serbia but the actual number may be higher as Roma tend to hide their ethnic origin. Traditionally encouraged Roma behaviors include early endogamous marriages and high fertility. In Serbian Roma, rates of low birthweight have been increasing while estimates for infant and under five years old child mortality rates run three and four times higher than for non-Roma [25]. In comparison to non-Roma, Roma children are at risk of not achieving their developmental potential because of poor nutrition and parental care, which may negatively influence life outcomes, leading to an intergenerational cycle of poverty and poor development [7,26]. Yet, there exist some health differentials among Roma children themselves, suggesting that some children are especially vulnerable [27]. Despite this variance, to date, no research has directly assessed variability in Roma mothers’ parental care, and whether, after controlling for birthweight, child wantedness accounts for some of the biases in parental investment. Using data from UNICEF 2014 Multiple Indicator Cluster Survey 5 (MICS5) for Serbian Roma settlements, this study aimed to characterize the relationship between low birthweight, child wantedness and bias in parental investment.

## 2. Materials and Methods

### 2.1. Study Design and Sample

The present study was performed as a secondary data analysis of the MICS5, a public use dataset, administered in Serbia in 2014, and carried out by the Statistical Office of the Republic of Serbia with support from UNICEF (available at http://mics.unicef.org/surveys). The survey sample is designed to provide estimates of maternal and child health indicators at the national and regional level, and separately for Roma communities. MICS-5 capture both anthropometric and early child development data along with basic information on mothers, caregiving practices for young children, and household wealth. The MICS-5 includes a specific series of questions that capture several domains of child development and parental engagement. Roma mothers (or caregivers) were asked to provide information on their children’s age, gender, birth order, care and feeding practices, and mother–child interaction. Weight at birth and vaccination records were obtained from health cards, health facilities and mothers’ recall. Mothers with singleton birth in the past two years prior to the survey, in a marriage or with partners in unregistered unions, were included in the study. There were 584 children aged 0–24 months weighed at birth.

### 2.2. Measures of Parental Investment

Two base level investments, breastfeeding practices and child immunization status, and one surplus resource, the quality of mother–child interaction, were used as separate measures of parental investment. These measures capture different domains of parental investment; at the base level, relatively low cost (base) investments are essential for greater chances of offspring survival and health, and may include in utero nutritional transfers, practices such as protection, breastfeeding, food allocation, preventing and health seeking behaviors [28,29]. In contrast, parents can also choose to provide surplus resources such as investing in their children’s education or other investments that may enhance their child’s wellbeing besides those necessary to ensure survival alone [19,30]. The latter may depend more on the parents’ own availability of resources, but both types of investments may have immediate and long-term benefits at the same time [23,31].

Breastfeeding practices were self-reported by the mother, and had two modalities: whether the child has ever been breastfed, and first nursing after birth measured in time units. The benefits of breastfeeding for child survival and health are well known, positively affecting cognitive development and providing protection against diseases appearing later in development; putting newborn babies to the breast within the first hour after birth provides them with the best chance to survive, thrive and develop to their full potential [32]. Thus, breastfeeding may be the most direct, base level measure of parental investment [33].

Immunization status refers to whether a child received full vaccinations against common childhood diseases. Immunization greatly reduces disease, disability, and death, benefiting the children but also society as a whole [34]. According to the Republic of Serbia’s required immunization calendar, full immunization is defined as: the bacille Calmette–Guérin (BCG) vaccine against tuberculosis; three doses of diphtheria–tetanus–pertussis (DTP); three doses of polio vaccine; three doses of Hepatitis B (HepB); three doses of Hemophilus influenza type B (Hib); one dose against measles, mumps, and rubella (MMR) [25]. All children born in a hospital receive BCG and a first dosage of HepB before discharge, while the rest of the vaccines are provided by local health facilities to all children free of charge. The immunization status of children was obtained from mothers’ recall, vaccination cards and medical records from local health facilities.

Mother–child interaction, reported by the mother, refers to the types and number of activities a mother engaged in with her child during the previous three days. Six activities—reading books or looking at picture books; telling stories to the child; counting or drawing with the child; singing songs/lullabies; taking the child outside the home, into a yard, or park; playing with the child—are the common proxies for quality parent–child interaction, regarded as essential for early cognitive development and socio-emotional wellbeing [35]. The total score of activities ranges from 0–6 points; in this study, the number of activities was grouped into three categories: low engagement (0–2 activities), moderate engagement (3–4 activities), and high engagement (5–6 activities).

### 2.3. Maternal and Child Variables

The independent maternal variables were age, literacy (can read the whole sentence/basic literacy or can read only part of the sentence/functionally illiterate) and household wealth, based on MICS wealth index comprised of a list of assets measured at the household level and made into five ordered quintiles, i.e., the first being lowest wealth. The child variables were gender, birth order, birthweight (low birthweight at <2500 g and normal birthweight at >2500 g) and whether the child was wanted, assessed by the question if the pregnancy with a particular child was desired at that exact time. Mistimed pregnancy (wanted to get pregnant later) was not included as data were available for less than 10% of women.

### 2.4. Statistical Analyses

To handle missing data, pairwise deletion was used, resulting in an uneven number of cases (N).

Descriptive statistics and Chi-square tests with Yates’ Correction for Continuity, Fisher and *t*-tests were used to describe and detect differences across variables based on Roma children wantedness and birthweight. To detect the effects of unwanted pregnancy on the risk of low birthweight, a hierarchical binary logistic regression was conducted and included variables based on the literature review and their association with low birthweight in a previous study [27]. Predictor variables were mother’s literacy (dichotomous: 1—literate, 0—illiterate), whether the child was wanted (dichotomous: 1—yes, 0—no), child’s gender (1—female and 0—male), mother’s age at first reproduction (continuous) and whether a mother ever had a child who later died (dichotomous: 1—yes, 0—no), as a proxy for child mortality. Controlled variables were mother’s age (continuous), birth order (categorical, dichotomous: third or fourth born), and household wealth (categorical: 1st–5th quintile). Child’s weight at birth was coded as a dummy variable (1 > normal birthweight and 0 < low birthweight). Controlled variables were entered into the model in the first step, followed by the predictor variables in the second. Data were available for 535 children aged 0–24 months.

To determine whether a child’s wantedness could predict measures of parental (mother’s) investment, three separate regressions were performed. To predict breastfeeding practices and immunization status, two separate hierarchical binary logistic regressions were run, correcting for the influence of birthweight and several other independent variables. Control variables were age of the mother (continuous), household wealth (categorical: 1st–5th quintile), birth order (categorical, dichotomous: third or fourth born) and child’s weight at birth (coded as a dummy variable: 1 > 2500 g and 0 < 2500 g). Predictor variables in the two models were child’s gender (1—female and 0—male), mother’s literacy (dichotomous: 1—literate, 0—illiterate) and whether the child was wanted (dichotomous: 1—yes, 0—no). In both regressions, in the first step, the control variables were entered in the model, while the second step involved inclusion of the predictor variables. Dependent variable breastfeeding practices utilized two models, one for each modality: in the first model, the dependent variable was whether the child has ever been breastfed, available for 537 children (1—yes, 0—no), and in the second, the dependent variable was first nursing after birth measured in time units (1—immediately breastfed, 0—breastfed later), and included 509 children who were in the same room with their mothers after birth.

Immunization status of the children was coded as a dummy variable (1—full immunization, 0—partial immunization). Roma children younger than 12 months were excluded from the analyses, as they were too young to have received the full immunization coverage; also excluded were the children whose mothers provided immunization status from recall, thus leaving 246 children.

To assess the quality of mother–child interaction, a multinomial logistic regression was conducted, where the quality of interaction was divided into three categories: (1) low engagement, (2) moderate engagement and (3) high engagement. Predictor variables were age of the mother, literacy, household wealth, child’s birth order, weight at birth, gender, and whether the child was wanted, all coded as per the previous models. Data were available for 293 children aged 12–24 months.

IBM SPSS Statistics V22. was used to perform the analyses.

## 3. Results

### 3.1. Descriptive Statistics

According to Roma mothers, almost all children (98%) were born in a hospital. For over 35% of Roma children, mothers were unable to produce a birth certificate, even though they claimed that birth registration had taken place.

Average weight at birth for Roma children aged 0–24 months was 3086 g (M = 3.07; SD = 0.77). Of these, 15.8% of children had low weight at birth (<2500 g) while 84.2% had normal weight. Among children aged 12–24 months, 14.4% (47) had low weight at birth and 85.6% (280) had normal weight. The mothers of low and normal birthweight children were not different in terms of key sociodemographic variables including age, literacy, household wealth, and parity (data not shown). Children with low and normal birthweight did not differ in terms of gender, birth order, immunization status and quality of mother–child interaction. Low birthweight children were on average older (M = 18.15; SD = 3.78) than normal birthweight children (M = 17.87; SD = 3.70), but the difference was not significant (t(325) = 0.47; *p* = 0.64). Statistically significant differences were found for breastfeeding practices—whether the child has ever been breastfed (Fisher’s two tailed test performed, *p* < 0.001), and first nursing after birth (Chi-square with Yates’ Correction for Continuity performed, χ2(1, N = 554) = 9.91, *p* < 0.001, φ = 0.13); low birthweight children were breastfed less and later on after birth than normal birthweight children. In addition, based on birthweight cut-off, there was a statistically significant difference between wanted and unwanted children, but the effect size was small (Chi-square with Yates’ Correction for Continuity χ2(1, *n* = 584) = 3.71, *p* = 0.05, φ = 0.08).

For children 0–24 months, 14.2% of mothers reported that the pregnancy with a particular child at the time was unwanted. Almost the same ratio was found among children from 12–24 months: 14.6% were unwanted. In total, 21% of children were both unwanted and born with low birthweight.

### 3.2. Differences in Sociodemographic and Parental Investment Measures Based on Child Wantedness

The sociodemographic and parental investment measures and differences based on Roma children wantedness are summarized in Table 1.

There was an excess of male vs. female children, with boys being more wanted than girls (χ2(1, *n* = 597) = 6.65, *p* = 0.01, φ = −0.11) (see Table 1). The difference in birthweight among unwanted and wanted children was significant but of small size effect (χ2(1, *n* = 584) = 3.71, *p* = 0.05, φ = 0.08). There were no differences in birth order, whether the child was ever breastfed and immunization status. The majority of children were fourth born and the rest were third born. Most children were breastfed but only one-quarter of the children had received full immunization, the highest coverage being for BCG and HepB, administered in a hospital immediately after birth (over 94%). Children with full immunization status were older (M = 19.47; SD = 3.29) than children with partial immunization (M = 17.37; SD = 3.71), and the difference was significant (t(244) = −3.99; *p* < 0.001). In regard to first nursing after birth, wanted children were breastfed straight after birth more than unwanted children (χ2(1, *n* = 565) = 11.99, *p* < 0.001, φ = 0.15).

The Roma mothers were relatively young, with an average age of 24 (range 15–44); mothers of wanted children were on average younger than mothers of unwanted children and the difference was statistically significant (t (595) = −2.14, *p* = 0.03). In spite of their relatively young age, the fertility of the Roma mothers was high, with four (SD = 0.26) children on average; the majority of mothers received antenatal care (96%), with an average of six times during a particular pregnancy, but only about 6% used modern methods of contraception. Most came from the two poorest quintiles. Level of basic literacy was low, and the difference in child wantedness varied with mother’s literacy skills (χ2(1, *n* = 549) = 4.26, *p* = 0.04, φ = −0.09). There was no difference in the quality of mother–child interaction. Most mothers engaged moderately in activities with their children: the average level of engagement was 3.29 (SD = 1.44), while 6% (20) of mothers had no engagement whatsoever (0 activities). Frequencies for the activities with a child were not available, but only 14% of Roma mothers engaged in book reading, the majority (60%) engaged in storytelling, singing songs (74%), playing with (86%) and taking the child outside (77%), while only 16% named counting or drawing with the child.

### 3.3. The Effects of Child Wantedness on the Risk of Low Birthweight

The effects of child wantedness on the risk of low birthweight are shown in Table 2.

Only the second model with predictor variables was significant (χ2(11) = 20.64, *p* = 0.04), implying that the predictor variables explained the dependent variable, i.e., low birthweight among Roma mothers. The model explained between the 3.8% (Cox and Snell R2) and 6.6% (Nagelkerke R2) variance of the dependent variable. Children who were wanted had higher odds of weighing more than 2500 g at birth in comparison with children who were not wanted (OR = 2.42; 95% CI = 1.29–4.56; *p* = 0.01). Children born to mothers who experienced child death had lower odds of having more than 2500 g at birth in comparison to those born to mothers with all-surviving children (OR = 0.31; 95% CI = 0.11–0.91; *p* = 0.03). Children born to mothers with an older age at first reproduction had lower odds of having more than 2500 g at birth in comparison with children born to mothers with earlier age at first birth (OR = 0.91; 95% CI = 0.82–1.00; *p* = 0.04).

### 3.4. The predictors of Parental Investment: Breastfeeding Practices, Immunization Status and Quality of Mother–Child Interaction

The predictors of parental investment—breastfeeding practices, immunization status and quality of mother–child interaction—are shown in Table 3.

Both models, with controlled and independent variables, explaining whether the child has ever been breastfed, were statistically significant (χ2(7) = 26.39, *p* < 0.001 and χ2(10) = 28.77, *p* < 0.001, respectively). Control variables explained between 4.8% (Cox and Snell R2) and 14.0% (Nagelkerke R2), and independent between 5.2% (Cox and Snell R2) and 15.2% (Nagelkerke R2) variance of the dependent variable. After correcting for the influence of the control variables, the independent variable that explained whether the child has ever been breastfed was birthweight. Children born with more than 2500 g at birth had higher odds of being breastfed when compared to low birthweight children (OR = 6.47; 95% CI = 2.88–14.58; *p* < 0.001).

Regarding first nursing after birth, only the full model was significant (χ2(10) = 20.61, *p* = 0.02), thus, independent variables significantly contributed to the explanation of the dependent variable: between 4.0% (Cox and Snell R2) and 5.6% (Nagelkerke R2) of the variance. Wanted children had higher odds of being breastfed immediately after birth when compared to the unwanted children (OR = 2.41; 95% CI = 1.40–4.12; *p* < 0.001), as well as children born with birthweight of more than 2500 g (OR = 1.83; 95% CI = 1.07–3.13; *p* = 0.03).

With regard to immunization of children, only the model with independent variables was significant (χ2(10) = 21.79, *p* = 0.02) and explained between 5.7% (Cox and Snell R2) and 10.3% (Nagelkerke R2) of the variance of the dependent variable. After correcting for the influence of controlled variables, the independent variable of whether the child was wanted explained a child’s immunization status: wanted children had higher odds of being fully vaccinated than unwanted children (OR = 5.56; 95% CI = 1.61–19.19; *p* = 0.01). Out of controlled variables, birthweight and birth order contributed to the explanation: normal birthweight and fourth born children had lower odds of being fully vaccinated than low birthweight and third born children (OR = 0.43; 95% CI = 0.19–0.96; *p* = 0.04, and OR = 0.21; 95% CI = 0.08–0.56; *p* < 0.001, respectively).

The regression model for the quality of mother–child interaction was significant (χ2(20) = 35.30; *p* = 0.02; Pearson χ2(442) = 413.59; *p* = 0.83; Deviance χ2(442) = 321.50; *p* = 1.00). Predictor variables explained between 11.4% (Cox and Snell R2) and 14.7% (Nagelkerke R2) of mother–child interactions. Mother’s household wealth significantly contributed to the model (χ2(8) = 22.07; *p* = 0.01). Thus, mothers from the richest homes, compared with mothers from the poorest homes, were more likely to have higher engagement than low engagement with the child (OR = 0.07, CI = 0.01–0.73, *p* < 0.001). Likewise, mothers from average wealth households were more likely to have higher engagement than low engagement with the child, when compared with mothers from the poorest homes (OR = 0.16, CI = 0.03–0.76, *p* = 0.02). Similar gradients are seen between moderate and high engagement, in that mothers from average and rich households were more likely to have higher engagement than moderate, when compared with mothers from the poorest homes (OR = 0.34, CI = 0.17–0.93, *p* = 0.04, and OR = 0.18, CI = 0.06–0.54, *p* < 0.001, respectively).

## 4. Discussion

This study examined the associations between low birthweight, child wantedness and biased parental investment among Serbian Roma mothers. Previous studies found inconsistent relationships between child wantedness and low birthweight [36,37,38,39], but in this study, after adjusting for potential confounding factors, child wantedness predicted poor birth outcome. As weight at birth is a measure of parental investment during pregnancy, this implies that Roma mothers’ investment in utero was lower for unwanted children, i.e., child outcome correlated with the investment itself [20,40]. Consistent with previous studies of maternal reproductive history, having had a child who died and an older age at first reproduction were significantly associated with infant low birthweight [41].

The relationship between child wantedness, birthweight and Roma mothers’ parental investment is not straightforward. Breastfeeding varied with birthweight and child wantedness. In addition to its protective effects, breastfeeding is also a significant building block of the mother–child bond, as for many women, breastfeeding induces strong positive feelings associated with maternal hormones [42,43]. Breastfeeding thus may be an indicator of a mother’s willingness and capacity to invest in her child. In many Eastern European Roma communities, the practice of breastfeeding remains an integral part of the Roma mothers’ cultural identity [44]. In this sample, the majority of Roma mothers breastfed their children, but discriminated against low birthweight children. After adjusting for potential confounders, child wantedness predicted first nursing after birth; the other factor significantly associated with first nursing was birthweight. In line with other studies, unwanted and low birthweight children received less investment than their counterparts [45,46].

Whether the child was wanted or not was the key explanatory variable regarding immunization status, as also observed in other studies: wanted children had higher odds of being fully vaccinated than unwanted children [47]. However, and contrary to other studies, birthweight had an opposite outcome, in that Roma mothers boosted their investment in children with low birthweight [12,48]. In contrast to the Roma, mothers in populations with very high levels of investment in offspring tend to increase investment in offspring in poorer conditions [33]. In situations where children are failing to thrive, mothers might try to increase investment in order to try to mitigate children’s health status. Investment in childhood immunization is very low among Roma, albeit that the Serbian vaccination schedule is offered to all children free of charge. Across Serbian Roma settlements, for children up to 35 months of age, more than 56% are not covered by the recommended immunization, while for the younger children (up to their first birthday), the number is even higher–87% [25]. This pattern was also observed in the current sample: older Roma children received more vaccination coverage than younger children, e.g., the fourth born; given that low birthweight children were on average older than normal birthweight children, this may be another possible explanation for the greater vaccine coverage for the low birthweights. Parental investment tends to be higher for older offspring and reduced with each additional birth; cross-culturally, a child’s age is correlated with reproductive value but also with mortality, which is higher for the youngest children [49].

Numerous studies across Europe have found vaccination uptake to be lower among Roma compared to non-Roma [50]. In Central and Southeastern Europe, this difference does not appear to be entirely explainable by the more adverse socioeconomic status of the Roma, or by rejection of immunization based on cultural or religious grounds [51]. Given Roma segregation and low education status, it is likely that unawareness of need for immunization and the general passiveness of the Roma contribute to low vaccination coverage. This is indicative, for example, in the fact that the highest vaccine coverage among Roma children is for BCG and HepB, given to all babies in a hospital, a situation where Roma mothers do not have to act on their own, and in contrast to other vaccines, where parents have to present their children to health providers in order to receive coverage. In other instances, Roma parents tend to react to various incentives offered: Serbian health officials, from time to time, organize campaign vaccinations in Roma settlements, where they provide packages of humanitarian and other help and vaccinate children at the same time [52].

Gradients in household wealth explained mother–child interaction, while birthweight and wantedness were insignificant. In numerous studies, variation in parental investment, examined with different measures, varied according to maternal socioeconomic position [53,54]. Given the general poverty of Roma, the significance of wealth for children in mother–child interaction, may simply reflect a result of being born into families that have more resources to invest, such that the variation in surplus investments was primarily shaped by access to resources [55]. Many Roma children grow up in unstimulating home environments, lacking books and toys [26] and the activities with children in the home, such as reading books or teaching them about letters and numbers, require not only actual books but also basic literacy and numeracy skills, which many Roma women do not possess. Unlike other studies, maternal age had no influence on parental decisions regarding investment [29], but the sample of Roma mothers was age-homogenous and may influenced the lack of significance. Gender preference favoring girls among Roma was significant in previous studies [56,57] but had no effect in this study.

Among Roma mothers, unwanted children were at greater risk of having low birthweight. Both child wantedness and birthweight influenced mothers’ base level investments in children, while surplus investment varied with access to resources. The base level investments have well-established correlations with children’s health outcomes and are among the most generally recommended goals of public health campaigns [58]. As with other life-history decisions, there may be costs as well as benefits of parental investment, and those costs fall on the development of each individual child [59]. For many Roma mothers, the stress of poverty and lack of reliable income limit them to focus their time and energies on parenting as they are constantly struggling to survive [60]. Most likely, Roma mothers, just like any other parent on average, have developed strategies to invest in their children based on their own experiences, social position and sensitivity to environmental factors [61]. Among the complex negative factors accompanying underdevelopment, unwanted children may be the most serious, as being unwanted, in addition to low birthweight and high neonatal mortality risk, may have a long-lasting effect on a child’s development, and is associated with adverse life cycle outcomes [62]. Very few Roma women use modern contraceptives, but the majority cease reproduction in their late twenties, with an average of four surviving children, implying that together with their partners, they make a deliberate decision to stop reproducing after the desired number of children is reached [63]. There was no difference in wantedness between the third and fourth born children, suggesting that factors other than desired number of children might influence child wantedness. Future studies should examine the role of female kin support on unwanted childbearing, as having supportive kin may not only buffer against life obstacles but also be beneficial for reproductive success [64].

This study contributed new evidence from the Serbian Roma national dataset about the associations between child wantedness, birthweight and parental care, adding to the literature about parental investment and child outcomes in poor ethnic minority populations. The present study included several limitations. The study design was cross-sectional, limiting causal inference. The MICS-5 datasets provide limited range of mostly self-reported variables, thus allowing for potential biases. Reports of child wantedness were likely influenced by the presence of the child [65]. Birthweight data included a cut off for low vs. normal, but not the actual weight, gestation, or underlying health issues, which prevented inferring of more possible causal influences. Other potential confounders, such as mother’s height, health status, medical complications of pregnancy (hypertension or diabetes), and history of prior preterm or low birthweight births, were not collected. Measures accounted only for surviving children, and if disadvantaged children suffered higher mortality, this would also skew the results [66,67]. Furthermore, even though MICS program is a nationally representative and internationally standardized household survey, the methodology and a number of survey measures may not be entirely suitable for the Roma culture. The MICS-5 for the Serbian Roma relied on contracted amateur local interviewers; the Roma are a hard-to-reach traditional culture, largely reluctant to give information to outsiders and it usually takes a considerable amount of time to gain access and especially trust to negotiate a researcher’s presence and partial acceptance [68]. Some indicators of parental care measured may not be entirely typical for Roma nor represent all possible ways that mothers invest in their children: for instance, a proximal style of parenting, consisting primarily of body contact and body stimulation but without much verbalism, may be a more appropriate measure of mother–child interaction among Roma than the one mentioned, thus, new research should focus on developing a new set of indicators culturally relevant to the Roma [69].

Reducing unwanted childbearing is important for improving a child’s health and requires an understanding of the reasons behind it, which go beyond securing a minimum desired number of live children in a context of high mortality [66]. With the high rates of infant and child mortality among Roma, investments in children’s health should be prioritized within the family, where maternal bias in parental investment may contribute to their children’s health disparities.

## Figures and Tables

**Table 1:** Sociodemographic and parental investment measures distribution based on Roma children wantedness.

	Was the Child Wanted		*p* *	N (%)
	No	Yes		
Gender, N = 597, N (%)				
Male	34 (40.0)	282 (55.1)	0.01 **	316 (52.9)
Female	51 (60.0)	230 (44.9)		281 (47.1)
Child weight at birth N = 584, N (%)				
<2.5 kg	19 (22.9)	73 (14.6)		92 (15.8)
>2.5 kg	64 (77.1)	428 (85.4)	0.05 **	492 (84.2)
Mother’s literacy, N = 549, N (%)				
illiterate	20 (25.6)	178 (37.8)	0.03 **	198 (36.1)
literate	58 (74.4)	293 (62.2)		351 (63.9)
Birth order, N = 597, N (%)				
3.	10 (11.8)	35 (6.8)	0.11 **	45 (7.5)
4.	75 (88.2)	477 (93.2)		552 (92.5)
Child ever been breastfed, N = 597, N (%)				
no	7 (8.2)	25 (4.9)	0.19 ****	32 (5.4)
yes	78 (91.8)	487 (95.1)		565 (94.6)
First nursing after birth (unit), N = 565, N (%)				
later	38 (47.5)	137 (28.2)	0.001 **	175 (31.0)
immediately	42 (52.5)	348 (71.8)		390 (69.0)
Immunization status, N = 240, N (%)				
partial	31 (83.8)	148 (72.9)	0.16 **	179 (74.6)
full	6 (16.2)	55 (27.1)		61 (25.4)
Mother–child interaction, N = 330, N (%)				
low or no engagement	1 (2.1)	27 (9.6)	0.13 **	28 (8.5)
moderate engagement	35 (72.9)	206 (73.0)		241 (73.0)
high engagement	12 (25.0)	49 (17.4)		61 (18.5)
Household wealth, N = 597, n (%)				
Poorest	23 (27.1)	155 (30.3)	0.21 **	178 (29.8)
Second	23 (27.1)	125 (24.4)		148 (24.8)
Middle	11 (12.9)	95 (18.6)		106 (17.8)
Fourth	9 (10.6)	67 (13.1)		76 (12.7)
Richest	19 (22.4)	70 (13.7)		89 (14.9)
Mother’s age, N = 597, mean (SD)	25.01 (5.69)	23.69 (5.21)	0.03 ***	23,99 (5.44)

* *p* = ≤ 0.05; ** Chi-square with Yates’ Correction for Continuity; *** *t*-test; **** Fisher’s test.

**Table 2:** Predictors of low birthweight.

			Weight at Birth
			OR (95%CI)
Mother	Intercept		1.88
	Age		1.08 (1.01, 1.15)
	Mother’s literacy	Literate	0.76 (0.43, 1.33)
	Was the child wanted	Yes	2.42 (1.29, 4.56) *
	Ever had a child who later died	Yes	0.31 (0.11, 0.91) *
	Age at first reproduction		0.91 (0.82, 1.00) *
	Household wealth	Second	1.04 (0.55, 1.20)
		Middle	0.97 (0.47, 2.01)
		Fourth	1.48 (0.62, 3.55)
		Richest	2.16 (0.83, 5.66)
Child	Gender	Female	1.20 (0.73, 1.98)
	Birth order	4th	1.67 (0.73, 3.80)

* *p* = ≤ 0.05.

**Table 3:** Predictors of parental investment.

			Breastfeeding Practices		Immunization Status	Mother–Child Interaction	
			Child Ever Been Breastfed	First Nursing (Unit)		Low Engagement	Moderate Engagement
			OR (95%CI)	OR (95%CI)	OR (95%CI)	OR (95%CI)	OR (95%CI)
Mother	Intercept		1.25	1.30	0.09		
	Age		1.01 (0.93, 1.09)	0.99 (0.95, 1.02)	1.03 (0.97, 1.09)	0.94 (0.85, 1.05)	1.00 (0.94, 1.07)
	Mother’s literacy	Literate	1.59 (0.63, 4.00)	0.89 (0.57, 1.40)	1.06 (0.52, 2.16))	0.54 (0.18, 1.65)	1.86 (0.61, 5.71)
	Was the child wanted	Yes	1.90 (0.69, 5.22)	2.41 (1.40, 4.12) *	5.56 (1.61, 19.19) *	4.34 (0.49, 38.67)	1.16 (0.48, 2.81)
	Household wealth	Second	1.85 (0.51, 6.74)	0.79 (0.46, 1.34)	2.04 (0.86, 4.85)	0.45 (0.12, 1.65)	0.72 (0.28, 1.90)
		Middle	0.59 (0.18, 1.91)	0.74 (0.41, 1.33)	0.91 (0.32, 2.63)	0.16 (0.03, 0.76) *	0.34 (0.17, 0.93) *
		Fourth	0.32 (0.09, 1.08)	0.62 (0.31, 1.21)	1.54 (0.52, 4.52))	0.00 (0.00,0.00)	0.80 (0.23, 2.79)
		Richest	0.60 (0.15, 2.42)	1.04 (0.52, 2.10)	2.32 (0.79, 6.77)	0.07 (0.01, 0.73) *	0.18 (0.06, 0.54) *
Child	Birthweight	>2.5 kg	6.47 (2.88, 14.58) *	1.83 (1.07, 3.13) *	0.43 (0.19, 0.96) *	0.69 (0.16, 3.07)	0.66 (0.23, 1.88)
	Gender	Female	1.26 (0.56, 2.86)	0.94 (0.63, 1.39)	1.33 (0.72, 2.46)	1.80 (0.64, 5.11)	1.49 (0.76, 2.94)
	Birth order	4th	1.79 (0.53, 6.04)	0.90 (0.42, 1.92)	0.21 (0.08, 0.56) *	1.92 (0.42, 8.83)	1.96 (0.76, 5.04)

* *p* ≤ 0.05.
